# Theory of Peaceful End of Life: Analysis and Evaluation Using the Whall Framework

**DOI:** 10.1002/hpm.70041

**Published:** 2025-11-30

**Authors:** Romel Jonathan Velasco Yanez, Ana Luiza Almeida De Lima, Marcos De Oliveira Lopes, Viviane Martins Da Silva, Ana Fátima Carvalho Fernandes, Maria Célia Freitas

**Affiliations:** ^1^ Department of Nursing Federal University of Ceará Fortaleza Brazil; ^2^ Postgraduate Program in Clinical Care in Nursing and Health Ceará State University Fortaleza Brazil

**Keywords:** end‐of‐life, nursing care, nursing education research, nursing theory, palliative care

## Abstract

**Aim:**

This study aims to analyse and evaluate the theory of peaceful end of life to determine its relevance and applicability in nursing research, education and practice, particularly in the areas of palliative care and end‐of‐life care.

**Methods:**

We conducted a theoretical‐reflexive study using specific criteria for analysing and evaluating middle‐range theories as proposed by Ann Whall (2005).

**Results:**

The theory clearly outlines the dying and death process, connecting key concepts and essential statements that support a peaceful end of life. It also implicitly incorporates elements of the nursing metaparadigm. Additionally, the theory demonstrates adequate internal consistency and empirical adequacy.

**Conclusions:**

While the theory offers valuable guidance for nursing research, education and practice in palliative and end‐of‐life care, several information gaps were identified that require refinement for its optimal application. Further studies incorporating all the theory's concepts and statements are needed to confirm its testability, empirical validity and potential need for modifications.

**Implications for the profession:**

The theory of peaceful end of life has been widely applied in nursing, especially in the context of palliative care, in recent years. However, no previous critiques of the theory have employed a specific evaluation framework for middle‐range theories. This study presents the first formal critique using an appropriate evaluation framework. The findings could help nurses apply this theory more effectively in the care of patients at the end of life.

**Patient or Public Contribution:**

No patient or public contribution was involved in this study.

## Highlights

- Importance of evaluating nursing theories for research, education and practice.
- The theory of peaceful end of life can be widely applied in palliative and end‐of‐life care.
- Refinement is needed to address the identified knowledge gaps in the theory.

## 1 Introduction

The demographic shift brought about by increased life expectancy has generated significant public health issues. The transition in the epidemiological profile of the global population has led to a predominance of Non‐Communicable Chronic Diseases (NCDs), which account for over 70% of deaths globally. This translates to physiological deterioration, increased hospitalisations, and longer stays in hospital settings. Addressing NCDs requires substantial investment in financial resources, specialised healthcare services, palliative care (PC), and medical staff training [1].

This change in the population profile highlights the need to establish and plan a care model that supports the patient's illness process, as well as the death and dying process, with the aim of improving quality of life during both the illness and the end‐of‐life phase, as proposed by PC [2].

In this context, it is essential to understand that PC represents a philosophy of holistic and active care directed towards individuals of all ages who are severely suffering from serious illnesses, especially those nearing the end of life. Therefore, PC should be offered as early as possible to address and prevent suffering, even if the individual is not in the final stage of life [3].

To provide excellent PC that prioritises holistic care and comfort, it is recommended to adopt a multidisciplinary team approach, with nursing playing a key role as the first link in the triad formed by the team, the patient, and the family [4, 5, 6]. In this context, nursing theories have emerged to develop critical reasoning based on science, addressing the profession's interests and specificities, as well as the needs across diverse social contexts [7]. Therefore, it is necessary to evaluate the quality and relevance of these theories in clinical practice to establish their true scope, application, and potential gaps that need refinement [8], as well as their dissemination and discussion among nursing students.

In light of this scenario and the need for a theoretical foundation that supports the philosophy of PC, the Theory of the Peaceful End of Life (TPEL) [9] has gained prominence, as it advocates for safeguarding life in its fullness, ensuring that the ill individual and their family can experience moments of peace and well‐being during the terminal process. TPEL is a middle‐range nursing theory (MRT) developed in 1998 by nurses Ruland & Moore [9] and is considered a reemerging theory, as it has gained increased relevance in the current context of PC discourse.

To bring originality to this study, we conducted a literature search for similar work and identified two studies that evaluate the TPEL [10, 11]. However, both studies used evaluation frameworks for grand theories which, in our view, may be less appropriate for evaluating MRTs. In this study, we propose using the framework developed by Ann Wall (2005) [12], which specifically addresses the analysis and evaluation of MRTs, modifying the guidelines used for analysing and evaluating grand nursing theories. Thus, our study aims to analyse and evaluate the TPEL using the model proposed by Whall.

## 2 Methods

This is a theoretical‐reflexive study based on the analysis and evaluation of the TPEL [9] using the framework proposed by Whall (2005). Whall's framework distinguishes three phases: (a) *basic considerations*, (b) *internal analysis and evaluation*, and (c) *external analysis and evaluation* [12]. Each phase provides guiding questions to objectively direct the process. To address each question, we developed the criteria shown in Table 1. The methodology of the analytical processes derived from the TPEL is detailed below:

**TABLE 1:** Whall's criteria for the analysis and evaluation of middle‐range theories.

Criteria for analysis and evaluation	Operational indicator
Basic considerations	
What are the definitions and relative importance of major concepts?	Definitions of the main concepts of the TPEL Relative importance of concepts and statements: — Theoretical substruction; sign matrix — Hardy's categorisation system
2. What is the type and relative importance of major theoretical statements and/or propositions?	
Internal analysis and evaluation	
What are the assumptions underlying the theory? What is the relationship to philosophy of science positions?	TPEL assumptions. Philosophical position of the TPEL.
2. Are concepts related/not interrelated via statements? Is there any resulting loss of information?	Interrelationships between concepts and statements. Identification of resulting information loss.
3. Is there internal consistency and congruency of all component parts of the theory?^a^	Criteria proposed by fawcett to evaluate internal consistency: Semantic clarity, semantic consistency, and structural consistency.
4. What is the empirical adequacy of theory?^a^ Has it been examined in practice and research and has it held up to this scrutiny?	Criteria proposed by fawcett to assess empirical adequacy: Traditional empiricism. Analysis of scoping review results.
External analysis and evaluation	
What is the congruence with related theory and research internal and external to nursing?	Analysis of scoping review results.
2. What is the congruence with the perspective of nursing, the domains, and the persistent questions?	Congruence of the TPEL with nursing science, metaparadigm, and important issues for the development of nursing.
3. What ethical, cultural, and social policy issues are related to the theory?	Relationship of the TPEL with ethical, cultural, and social policy issues.

^a^ These questions were replaced by the questions from the framework proposed by Fawcett.

### 2.1 Relative Importance: Theoretical Substruction

To determine the relative importance of the concepts and main statements of a theory, Whall suggests using the process of theoretical substruction [12, 13], defined as a strategy for critiquing a theory and research methodology. Through this process, the researcher identifies the main variables in a study, analyzes the levels of abstraction between variables, identifies hypothetical relationships between the variables, and connects the theoretical foundation of the study with the methodology [13].

The first step in theoretical substruction involved identifying the main definitions/concepts of the theory, that is, the central ideas that the theory aims to explain or predict. Once we identified the key concepts, we organised them in a hierarchical structure, placing the most fundamental concepts at the top and the more specific concepts at the bottom [13, 14].

The next step involved evaluating the relative importance of each concept. To do this, we assigned a score to each concept based on its centrality to the theory. The most fundamental concepts received the highest score, while the more specific concepts received a lower score [13]. We used a Likert‐type scale where 5 represents the most fundamental concepts, and 1 represents the non‐fundamental concepts.

Once the relative importance of each concept was established, the next step was to analyse the links between concepts. This involved examining how each concept relates to the others and identifying missing links or gaps in the theory [13]. For this analysis, we used the sign matrix. The sign matrix is a visual tool that allows for the organisation and categorisation of the concepts and statements of a theory. Each concept or statement is placed in a row or column of the matrix, and the relationships between them are indicated by cells in the matrix, identifying positive (+), negative (−), or unknown (?) relationships [15, 16].

### 2.2 Categorisation of Statements: The Hardy System

To establish the type of theoretical statements proposed in the TPEL, we used the Hardy system [17]. The Hardy system is used to evaluate the logical coherence of nursing theories and to identify possible gaps or inconsistencies in their structure through the categorisation of statements according to their logical level of complexity [17]. The categories include statements, postulates, propositions, hypotheses, axioms, laws, principles, and empirical generalisations.

### 2.3 Internal Consistency

To evaluate the internal consistency of a theory, Whall's analysis and evaluation framework [12] references consistent use of terms as a criterion. However, since Whall's framework does not provide a detailed methodology for this, we decided to evaluate the internal consistency of the TPEL using the methodological criteria established by Fawcett [15], who defines internal consistency as the harmony between the concepts that are part of a model or theory, that is, the coherence of the concepts used, their interdependence, and their ability to explain a phenomenon.

### 2.4 Empirical Adequacy

Similarly, Whall's framework [12] does not clearly define empirical adequacy or how to evaluate it. Therefore, we also used the criteria proposed by Fawcett for MRTs for its evaluation [15]. According to Fawcett, the criterion of empirical adequacy requires that the statements of a theory align with empirical evidence. If empirical data support the theoretical claims, it may be appropriate to tentatively accept these claims as reasonable or adequate. To assess this, Fawcett suggests using the ‘*traditional empiricism*’ approach, which requires that the concepts of a MRT be observable and the propositions measurable. This criterion is met when specific instruments or experimental protocols have been developed to observe the theoretical concepts [15].

To determine empirical adequacy, Fawcett recommends a thorough review of the findings from all studies based on the theory. Consequently, we conducted a scoping review to address both empirical adequacy and the external analysis and evaluation of the TPEL using Whall's criteria [12].

### 2.5 Procedures

We carried out the analysis and evaluation process in several stages, each conducted by two nurse researchers (RN1 and RN2) with experience in PC. Any discrepancies between them were discussed and mediated by two research professors (RP1 and RP2), who are experts in the construction, analysis, and evaluation of nursing theories. Before each stage, all four team members (RN1, RN2, RP1, and RP2) participated in a general meeting to discuss the methodological procedures to be followed. Next, the nurses (RN1 and RN2) conducted independent readings and added their observations to the analysis process. They then met to discuss their hypotheses collectively, and finally, RP1 and RP2 joined the discussion to provide their perspectives. All consensus decisions were reviewed and approved by the majority, and we followed this process for each stage.

### 2.6 Scoping Review

We conducted the scoping review following the methodological guidelines of the Joanna Briggs Institute (JBI) [18]. The review protocol was registered on the Open Science Framework (OSF) platform under the DOI: 10.17605/OSF.IO/3NHW7.

To formulate the review question, we used the PCC acronym (Population, Concept, and Context). The concept (C) refers to the use of the TPEL, and the context (C) refers to PC. It is important to note that we did not define a specific population. Therefore, our research question was: What evidence exists in the literature regarding the use of the Theory of Peaceful End of Life in palliative care?

We included all studies that met the inclusion criteria defined by the PCC acronym, with no restrictions on methodological design, date or language. We excluded articles that critically evaluated the TPEL, as well as the seminal article of the theory. To search for evidence, we consulted the databases Medline/PubMed, Embase, Scopus, and LILACS, using descriptors from controlled vocabularies (MeSH, Emtree, and DeSC), along with their synonyms and keywords, combined with the Boolean operators AND and OR. We also consulted nursing theory books and the references of the included studies. Grey literature was obtained from Google Scholar, Opengray, The ProQuest Dissertation & Theses Global, and The British Library. We created different search strategies for each database (Supporting Information S1), and these were reviewed by a librarian. The searches were conducted on June 22, 2023, and were limited to the languages Spanish, English, and Portuguese.

For the selection of studies, we used the software Rayyan Systems Inc. (RAYYAN) [19]. The selection process followed these steps; initially, we imported all retrieved articles into RAYYAN and removed duplicate articles. Next, we analysed all articles based on their titles and abstracts to identify those potentially eligible for inclusion. The articles identified in the previous phase were then read in full for a more thorough evaluation. At the end of this process, we obtained a list of studies that met the inclusion criteria and these were included in the review. Two reviewers conducted the review and selection process independently and blindly, and any decision conflicts were resolved by a third reviewer. The selection of grey literature was conducted exclusively by the lead researcher. We thoroughly reviewed all potentially eligible studies to ensure they met the inclusion criteria. Those that qualified were added to the final sample of included articles. The selection process is illustrated in the PRISMA 2020 flowchart [20] (Supporting Information S2).

We extracted and recorded the data in an extraction table adapted from another study [21]. Then, we analysed the data following Whall's evaluation framework. Additionally, we used descriptive statistics, including frequencies and percentages, to summarise the main variables identified in the included studies. We used different visual formats, such as graphs, charts, and tables, to present the results.

## 3 Results

The results of the analysis and evaluation of the TPEL are presented according to the stages outlined by Whall, as follows:

### 3.1 Basic Considerations of the Theory

The first step for analysis and evaluation is to answer the question: *what are the definitions and relative importance of major concepts?* Regarding the first question, the TPEL [9] presents five interrelated conceptual definitions, which are outlined below:

(a) C1. *Not being in pain* is defined as not experiencing pain. Pain has been described as an unpleasant sensory and emotional experience associated with actual or potential tissue damage, or described in terms of such damage. (b) C2. *Experience of comfort* is defined as the relief from discomfort, a state of tranquillity and peaceful satisfaction, and everything that makes life easy or pleasant. (c) C3. *Experience of dignity* is defined as being respected and valued as a human being. The notion of value is an important attribute of this concept. For a terminal patient, it implies being recognised and respected as an equal and not being subjected to anything that violates the patient's integrity and values. (d) C4. *Being at peace* involves the feeling of calmness, harmony and satisfaction. It means not being disturbed by anxiety, restlessness, worries or fear. (e) C5. *Closeness to significant others* is the feeling of connection with fellow human beings who care [9].

### 3.2 Relative Importance of the Concepts

**FIGURE 1:**
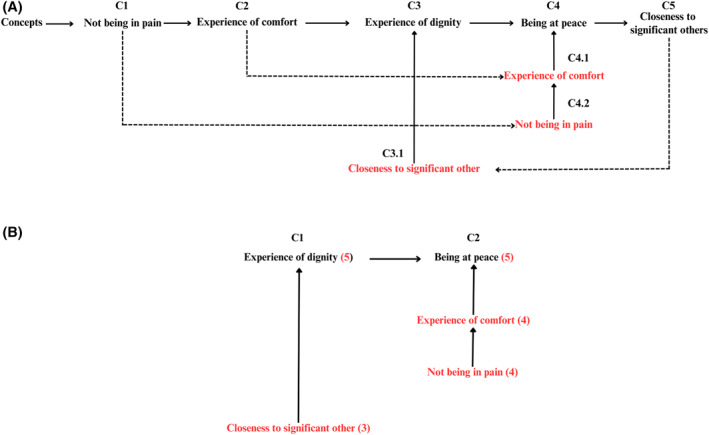
(A) Phase 1: theoretical substruction: Hierarchy of concepts. (B) Phase 2: theoretical substruction.

Once we identified the concepts, the next step was to establish their relative importance through the process of theoretical substruction. Figure 1a shows the hierarchical structure of the core concepts of the TPEL (C1–C5). We then logically and coherently grouped the concepts based on their mutual relationships, placing the most fundamental concepts at the top and the more specific concepts at the bottom (C4, C4.1, C4.2; C3, C3.1). In this way, we identified two hierarchies: ‘*being at peace*’ was identified as the most fundamental concept, as its attainment is directly related to the ‘*experience of comfort*’, that is, the sense of well‐being. This, in turn, cannot be achieved without proper ‘*pain relief*’. In the other hierarchy, the ‘*experience of dignity*’ is also a fundamental concept, influenced by ‘*closeness to significant others*’. We then evaluated the relative importance of each concept based on the scores assigned to each one. As a result, the ‘*experience of dignity*’ and ‘*being at peace*’ received a score of 5, as they were identified as the central concepts of the theory (Figure 1a–1b).

Finally, we established the relationships between the concepts of the TPEL using a sign matrix (Table 2). Most concepts showed positive relationships, indicating logical connections between them. However, we identified an unknown relationship between the concepts of ‘*not being in pain*’ and ‘*closeness to significant others*’, as well as between ‘*being at peace*’ and ‘*closeness to significant others*’. In other words, we could not determine to what extent being close to others might help relieve pain and contribute to ‘*being at peace*’.

**TABLE 2:** Sign matrix: Relationships between concepts.

Concepts	Not being in pain	Experience of comfort	Experience of dignity	Being at peace	Closeness to significant others
Not being in pain	x	+	+	+	?
Experience of comfort	+	x	+	+	+
Experience of dignity	+	+	x	+	+
Being at peace	+	+	+	x	?
Closeness to significant others	?	+	+	?	x

*Note:* Positive (+), negative (−), or unknown (?) relationships; There are no relationships among the same concepts (x).

Continuing with this initial stage, according to Whall, the next question to answer is: *what is the type and relative importance of major theoretical statements and/or propositions?* The TPEL presents six relational statements/declarations (Table 3), which, according to Hardy's logic levels, can be categorised as ‘*empirical generalisations*’. These statements arise from a process of consolidating the outcome criteria for the peaceful end‐of‐life standard for terminal patients, a standard developed by a group of clinical nurses in a surgical gastroenterology unit for the care of cancer and terminally ill patients [9]. Therefore, these are statements that are grounded on empirical observation of nursing practice. This aligns with Hardy's notion [17] that ‘*empirical generalisations*’ are assertions about data generally believed to be true, as they summarise empirical evidence and are thus closer to reality than hypotheses.

**TABLE 3:** TPEL Relational declarations/statements.

Relational declarations
D1. Monitoring and administering pain relief and applying pharmacologic and nonpharmacologic interventions contribute to the patient's experience of not being in pain.
D2. Preventing, monitoring and relieving physical discomfort, facilitating rest, relaxation and contentment, and preventing complications contribute to the patient's experience of comfort.
D3. Including the patient and significant others in decision making regarding patient care, treating the patient with dignity, empathy and respect, and being attentive to the patient's expressed needs, wishes and preferences contribute to the patient's experience of dignity and respect.
D4. Providing emotional support, monitoring and meeting the patient's expressed needs for anti‐anxiety medications, inspiring trust, providing the patient and significant others with guidance in practical issues, and providing physical presence of another caring person if desired contribute to the patient's experience of being at peace.
D5. Facilitating participation of significant others in patient care, attending to significant other's grief, worries and questions, and facilitating opportunities for family closeness contribute to the patient's experience of closeness to significant others or persons who care.
D6. The patient's experiences of not being in pain, comfort, dignity and respect, being at peace, closeness to significant others or persons who care contribute to peaceful end of life.

### 3.3 Relative Importance of Statements

**FIGURE 2:**
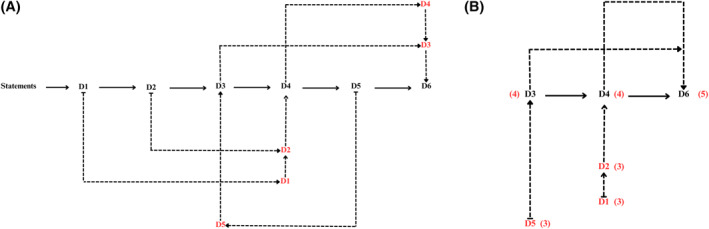
(A) Phase 1: theoretical substruction: Statement hierarchy. (B) Phase 2: theoretical substruction.

To conduct the theoretical substruction process for the statements, we followed the same steps as in the theoretical substruction of the concepts. Figure 2a illustrates the hierarchical structure of the statements in the TPEL (D1–D6). Initially, we identified two hierarchies (D3 and D4) which were closely related to the concept hierarchies, with each statement corresponding to a concept. However, there is a sixth statement that encompasses the previous five, which we identified as the fundamental statement (Figure 2a). We then assigned scores based on their centrality to the theory to define the relative importance of each statement. Accordingly, D6 received a score of 5 for being identified as the most fundamental statement of the theory. The other scores are shown in Figure 2a–2b.

Finally, we established the relationships between the statements of the TPEL using a sign matrix (Table 4). Most of the statements exhibit positive relationships with each other, indicating logical connections. However, there is an unknown relationship between statements D1 and D3, and between D1 and D4. Regarding the first pair (D1 and D3), although pain relief is theoretically implied in delivering dignity to a patient, the statements do not present semantic convergence to make such an inference. This situation is also reflected in the statements D1 and D4.

**TABLE 4:** Sign matrix: relationships between statements.

Statements	D1	D2	D3	D4	D5	D6
D1	x	+	?	?	+	+
D2	+	x	+	+	+	+
D3	?	+	x	+	+	+
D4	?	+	+	x	+	+
D5	+	+	+	+	x	+
D6	+	+	+	+	+	x

*Note:* Positive (+), negative (−), or unknown (?) relationships; There are no relationships among the same concepts (x).

### 3.4 Internal Analysis and Evaluation

For the internal analysis and evaluation of MRTs, Whall suggests answering the following questions: (a) *what are the assumptions underlying the theory? What is the relationship to philosophy of science positions?* To address these questions, we identified three assumptions in the TPEL: (1) the approach of a person to the end of life is a highly personal experience; (2) nursing care plays a crucial role in making this a peaceful experience; and (3) nurses can observe and interpret signals that reflect whether or not a patient is in a state of peace and intervene appropriately, even in cases where the patient cannot communicate verbally.

To analyse these assumptions concerning the philosophy of science position, it is important to note that Ruland & Moore do not explicitly report any philosophical stance or framework on which they based their theory. However, they mention that the theory was based on Donabedian's model of ‘*structure, process, and outcome*’ [9, 22], which has a consequentialist perspective, as it focuses on evaluating the quality of care in terms of its outcomes or consequences. Therefore, we can infer that the TPEL aligns with this consequentialist philosophical stance.

Additionally, in a text on Nursing Models and Theories [22], it is mentioned that the TPEL also drew on Brandt's ‘*theory of preference*’ [23], which focuses on ethical and moral decision‐making in situations where there are conflicts between the values and preferences of different individuals. According to Ruland & Moore, the quality of life at the end of life is based on the patient's preferences and values, as well as their ability to make informed decisions and participate in their own care. In this sense, the theory of preference emphasises the importance of considering the patient's preferences and values in nursing care at the end of life. Regarding the philosophical stance of this theory, it could be traced to a deontological or duty‐based perspective. However, it is important to note that in the original article [9], the authors do not mention the use of the theory of preference as part of their theoretical framework.

Continuing with the internal analysis, the next question to answer is: (b) *are concepts related/not interrelated* via *statements? Is there any resulting loss of information?* It is important to highlight that the main focus of the TPEL is not on the final instance of death itself but on providing support that contributes to a peaceful and meaningful life in the remaining time for patients and their significant others. It also stresses the importance of preparing for a calm and pain‐free death. The TPEL defines five concepts, as previously described, and six statements, with the first five statements corresponding to each concept (C1–D1; C2–D2; C3–D3; C4–D4; C5–D5), and the last statement (D6) summarising the interrelation with all the concepts.

With regard to the resulting loss of information, we identified that the TPEL does not present a specific concept for pain and comfort, with the authors providing definitions previously described in the literature. Therefore, since the experience of pain and comfort is intrinsic to each person and determined by multiple factors, it is necessary to consider their definitions in patients nearing the end of life. Another identified loss of information concerning the concept of ‘*not being in pain*’ is that, while the authors mention both pharmacological and non‐pharmacological interventions in their relational statement (D1), the involvement of a multidisciplinary team is not addressed in the TPEL. This is critical, as in most countries, nursing professionals are not authorised to prescribe medications. As a result, the scope of nursing interventions is limited by this omission.

The absence of an approach to patient spirituality can also be seen as a loss of information, as spirituality is essential to holistic and overall care. Additionally, although the TPEL emphasises throughout its constructs the importance of including family or significant others in ensuring a peaceful end of life for patients, it raises questions in scenarios where the patient lacks family support. In such cases, if a patient has no family or significant others, does this mean they will not experience a peaceful end of life? Moreover, within the ‘person’ metaparadigm [24], the TPEL does not clearly define which patients the theory applies to, considering factors such as age, mental conditions, among others.

The lack of a clear explanation regarding the ideal timing for implementing the TPEL is another potential loss of information, as this timing can vary depending on the patient's clinical conditions, a situation acknowledged by the theory's authors themselves. Finally, a perceived loss of information comes from the theory's title itself; while it includes the term ‘peaceful end of life’, the authors do not present a clear concept of what they consider to be a ‘peaceful end of life’, leaving room for varied interpretations of this phenomenon.

As part of the internal analysis and evaluation, Whall suggests questioning the internal consistency of the theory. To do this, we used Fawcett's criteria to answer the following questions: (c.1) *Are the context (philosophical claims and conceptual model) and the content (concepts and propositions) of the theory congruent?* (c.2) *Do the concepts reflect semantic clarity and semantic consistency?* and (c.3) *Do the propositions reflect structural consistency?* With regard to the first question, as mentioned earlier, the TPEL does not present an explicit philosophical stance, but it is congruent with the Donabedian model on which the theory is based [9, 22]. There is also congruence between its concepts and statements, as each statement corresponds to a concept.

In terms of semantic clarity, the TPEL meets this criterion, as it provides constitutive definitions for each concept, although, as previously mentioned, the concepts of ‘*not being in pain*’ and ‘*experience of comfort*’ are not original to the authors. As to semantic consistency, the TPEL also meets this criterion, as it consistently uses the same definition for each term/concept throughout the theory. Lastly, the TPEL exhibits structural consistency, as the links between the concepts are specified, and there are no apparent contradictions between its relational statements.

To conclude the internal analysis and evaluation of the TPEL, Whall suggests determining the empirical adequacy of the theory. To address this, we first examined the available evidence on the use of the TPEL. Below, we present the results of the scoping review we conducted.

We initially identified 62 relevant studies, of which 25 were excluded due to duplication. After evaluating the titles and abstracts of the remaining 37 studies, we excluded 23 for not meeting the inclusion criteria. We then proceeded to read the full texts of the remaining 14 articles, resulting in the exclusion of 4 additional studies, yielding a final sample of 10 studies. Additionally, we retrieved 507 documents of grey literature, of which 6 met the inclusion criteria and were included in our study. In summary, our final sample consisted of 16 studies, with all grey literature studies originating from scientific journals (Supporting Information S2).

Regarding the general characteristics of the studies, most were conducted between 2020 and 2022 (75%), in Brazil (43.7%), and employed a qualitative design (50%). Concerning the use of the TPEL, most studies utilised it for data analysis in light of the theory (50%), instrument development (12.5%), application of nursing care (12.5%), development of a situation‐specific theory (6.2%), explanation of care experiences (6.2%), and therapeutic intervention development (12.5%). Other characteristics are shown in Supporting Information S3.

After defining the available evidence, we used Fawcett's criteria to determine empirical adequacy by asking the following question: (*d*) *are theoretical assertions congruent with empirical evidence?* Our response is that the empirical data retrieved from the literature align with the TPEL's theoretical statements. Moreover, based on ‘*traditional empiricism*’ we observed that all the TPEL concepts are observable, and its statements are measurable. This is reflected in a study where an instrument was developed to assess nurses in terms of their level of practice in peaceful end‐of‐life care [25], as well as in other studies where a peaceful end‐of‐life care program was designed for terminally ill cancer patients receiving chemotherapy [26], and a quality improvement project was developed based on the concept of ‘dignity and respect experience’ [27].

Despite this, it is noteworthy that qualitative studies using the TFVP are limited to using it as a theoretical framework for data analysis. In this sense, it is significant that two studies [28, 29] addressed spiritual care in the death and dying process, yet the TPEL does not provide guidance for this approach, as previously mentioned.

### 3.5 External Analysis and Evaluation

Whall's framework suggests addressing the following questions for external analysis and evaluation: (a) *What is the congruence with related theory and research internal and external to nursing?* (b) *What is the congruence with the perspective of nursing, the domains, and the persistent questions?* and (c) *What ethical, cultural, and social policy issues are related to the theory?*

For the first question, we examined how well the TPEL aligns with the current body of nursing research and empirical evidence, as well as the extent to which the TPEL aligns with broader scientific knowledge from other disciplines. Based on the literature we reviewed, the TPEL shows congruence with internal nursing research, which is evident in several studies conducted within the field. For instance, eight studies used the TPEL as a theoretical framework to support their qualitative analyses [28, 29, 30, 31, 32, 33, 34, 35]; two studies developed an instrument based on the TPEL to evaluate nurses' level of practice in peaceful end‐of‐life care [25] and created a self‐administered questionnaire following the death of family members or friends [36]; two other studies used the TPEL as a guiding framework for providing nursing care at the end of life [37, 38]; one study used it to construct a situation‐specific theory on peaceful death in the Thai context [39]; two studies used it to develop intervention programs [26, 27]; and one study applied the TPEL to explain the experiences of Chinese nurses during the COVID‐19 pandemic [40]. As to the congruence of the TPEL with external nursing research, we did not find studies from other disciplines that utilised the theory.

Moving on to the second question, the TPEL shows congruence with the nursing perspective, as it addresses issues inherent to the nursing practice, such as quality of life during the death and dying process, and offer theoretical support for nursing professionals to base their practice on and provide a peaceful end of life. This alignment is also reflected in the TPEL's focus on the holistic care of patients at the end of life, considering not only their physical needs but also their emotional and social needs. The TPEL also aligns with the nursing domains (metaparadigm) [24], as its definitions and theoretical statements correlate with each concept of the metaparadigm (person, health, environment, and nursing). However, it is important to clarify that these concepts are only implicitly addressed by the theory. Finally, the TPEL is consistent with the persistent questions that continue to challenge the nursing profession in this field, such as peaceful death/dignified death [41], end‐of‐life care [42], hospice care [43], postmortem interventions [44], and grief management [45]—topics that are currently central to the development of PC.

In relation to the third question, the TPEL is directly related to ethical, cultural, and social policy issues which can be observed in the context of PC, where concepts like ‘euthanasia’, ‘orthothanasia’ and ‘dysthanasia’ have been the focus of current debates [46]. Euthanasia involves the act of painlessly inducing death to eliminate suffering, bringing about ethical and legal implications. On the other hand, orthothanasia is associated with a natural death without pain, distress, or suffering, while dysthanasia is the opposite of this concept.

The TPEL directly relates to orthothanasia, as it presents a theoretical framework oriented toward a peaceful death. Therefore, the ethical and cultural issues related to it derive from the political and health legislations of each country where nursing professionals practice. For example, one study adapted the TPEL to the Thai context to guide nurses in end‐of‐life decision‐making considering that, for Buddhists, the concept of death differs from that of western culture [39]. Similarly, two studies conducted in the Philippines [40] and China [25], respectively, highlighted the importance of culturally adapting the TPEL.

We did not find studies addressing the development of social policies based on the TPEL. However, it is important to mention that over the past two decades, the TPEL has been linked to advancements in PC policies, from the WHO's revision of the PC concept in 2002 [47], the WHA.6719 resolution in 2014 [48], to the current recognition by the same institution of the need to include PC into COVID‐19 response plans. Notably, within the PC scenario, each country develops its plans individually, as is the case in Brazil, Argentina, Colombia, and other countries in the region [49]. Despite these advancements, disparities in access to PC remain evident, with the WHO estimating that 40 million people need PC annually, yet only 14% of those who need it actually receive it [47, 50, 51].

## 4 Discussion

The TPEL is a middle‐range nursing theory that provides a theoretical framework for nursing professionals who care for patients at the end of life. Despite having been developed in the late 20th century, the ongoing debate on the importance of PC for patients with life‐threatening diseases positions it as a reemerging theory, alongside others such as Mishel's Uncertainty in Illness Theory [52], Kolcaba's Comfort Theory [53], and Lenz's Unpleasant Symptoms Theory [54], all of which are applicable in the palliative context.

In this study, we used the analysis and evaluation framework for MRTs proposed by Whall. Through this framework, we identified several information gaps within the TPEL that deserve special attention to improve and adapt the theory to the needs of patients at the end of life. Our findings partially differ from two other similar studies that analysed the TPEL [10, 11]. This discrepancy could be attributed to these studies using analysis frameworks designed for grand theories, such as those by Fawcett and Chinn & Kramer's, which are not fully suited for MRTs since they focus on more general and abstract aspects of a theory.

Among the information gaps we identified, the need to adjust the concepts of ‘*not being in pain*’ and ‘*experience of comfort*’ to the context of patients at the end of life, considering their particular needs, stands out [55, 56]. Likewise, we recognise the need to include concepts or relational statements that incorporate multidisciplinary teamwork [57] and spiritual care [58]. We also suggest revising the concept of ‘*closeness to significant others*’ to encompass patients who lack family support. Another information gap relates to the explicit identification of the ‘person’ metaparadigm, as it is unclear in which patients the TPEL can be applied, considering that age and mental conditions are determining factors. Lastly, we recommend specifying the timeframe in which the TPEL should be implemented, given that the remaining time for end‐of‐life care may vary according to clinical diagnosis, impacting the theory's applicability. This suggestion ties into the need to clearly define what is meant by a ‘peaceful end of life’.

Despite these limitations, the TPEL remains significant because it recognises that death is a natural part of life and that the focus of healthcare should shift as the patient approaches the end of life. Instead of continuing to aggressively treat the disease, it centres on providing relief and comfort to the patient, promoting closeness with loved ones, and preserving dignity. This can contribute to making the end of life more peaceful and less stressful for everyone involved. Moreover, the TPEL offers important guidance for nurses in caring for patients at the end of life.

Finally, it is also important to highlight the need to refine Whall's proposed framework for analysing and evaluating each criterion of MRTs in greater detail. While the framework provides a general structure for examining MRTs, it is essential to have more specific guidelines for each of the proposed criteria. Some suggestions could include the clear definition of measurable indicators for each criterion, the specification of the most appropriate data collection and analysis methods, and the inclusion of guidelines for interpreting the results obtained. These refinements would provide a stronger foundation for assessing the applicability and effectiveness of MRTs, allowing for greater confidence in their use in clinical practice and decision‐making in patient care.

## 5 Conclusions

The TPEL is a reemerging theory that provides explicit guidance on nursing care to achieve a peaceful death in patients at the end of life. This theory is relevant from both a social and theoretical perspective, aligning with the current context of PC. Although there is supporting evidence for its empirical adequacy, further research is needed to determine the empirical validity of each statement and the potential need for modifications. In summary, the TPEL plays a substantial role in guiding nursing research and practice in the context of end‐of‐life care. Its theoretical and social significance, supported by available evidence, demonstrates its utility in the field of PC. Nevertheless, refinements are needed in terms of scope, terminological consistency, and addressing identified information gaps. These steps will contribute to strengthening and advancing the TPEL, ultimately improving the care and well‐being of patients in the process of dying with dignity.

## Author Contributions

**Romel Jonathan Velasco Yanez:** conceptualisation, methodology, data curation, writing – original draft preparation, visualisation, investigation, writing – reviewing and editing. **Ana Luiza Almeida De Lima:** conceptualisation, methodology, data curation, writing – original draft preparation, visualisation, investigation, writing – reviewing and editing. **Marcos De Oliveira Lopes:** methodology, supervision. **Viviane Martins Da Silva:** methodology, supervision. **Ana Fátima Carvalho Fernandes:** data curation, writing – original draft preparation, writing – reviewing and editing. **Maria Célia Freitas:** data curation, writing – original draft preparation, writing – reviewing and editing.

## Funding

The authors have nothing to report.

## Ethics Statement

The authors have nothing to report.

## Conflicts of Interest

The authors declare no conflicts of interest.

## Supporting information


Supporting Information S1

Supporting Information S2

Supporting Information S3

## Data Availability

The authors have nothing to report.
