# Supplementary material for: Theory of Peaceful End of Life: Analysis and Evaluation Using the Whall Framework
Source: Int J Health Plann Manage. 2025 Nov 30;41(1):182–92. doi: 10.1002/hpm.70041 (PMC12794129; doi:10.1002/hpm.70041)
Supplement: Supplementary file 1 — Supporting Information S1 [file HPM-41-182-s002.docx]

| **Databases** | **Item searched** | **Retrieved (n)** |
| --- | --- | --- |
| MEDLINE/PubMed | (“Theory of the Peaceful End of Life” [Title/Abstract:~0] OR “Peaceful End of Life Theory” [Title/Abstract:~0] OR “Peaceful End of Life” [Title/Abstract] OR “Peaceful End-of-life Care” [Title/Abstract] OR “Peaceful End-of-Life Theory” [Title/Abstract:~0]) | 10 |
| Embase | ('theory of the peaceful end of life':ti,ab OR 'peaceful end of life theory':ti,ab OR 'peaceful end of life':ti,ab OR 'peaceful end-of-life care':ti,ab OR 'peaceful end-of-life theory':ti,ab) | 18 |
| Scopus | TITLE-ABS-KEY ((“Theory of the Peaceful End of Life” OR “Peaceful End of Life Theory” OR “Peaceful End of Life” OR “Peaceful End-of-life Care” OR “Peaceful End-of-Life Theory”)) | 27 |
| LILACS |  | 7 |
| **Organizations, Virtual Libraries and Websites and grey literature** | **Item searched** | **Retrieved (n)** |
| The British Library (UK) | (Peaceful End of Life Theory OR Peaceful End of Life) | 9 |
| Google Scholar | (Peaceful End of Life Theory OR Peaceful End of Life) | 135 |
| The ProQuest Dissertation & Theses Global | Peaceful End of Life Theory AND Palliative Care | 362 |
| Open Gray | (Peaceful End of Life Theory OR Peaceful End of Life) | 1 |

**Appendix 1. Supplementary file of search strategy**
