# Supplementary figures and images for: Theory of Peaceful End of Life: Analysis and Evaluation Using the Whall Framework
Source: Int J Health Plann Manage. 2025 Nov 30;41(1):182–92. doi: 10.1002/hpm.70041 (PMC12794129; doi:10.1002/hpm.70041)

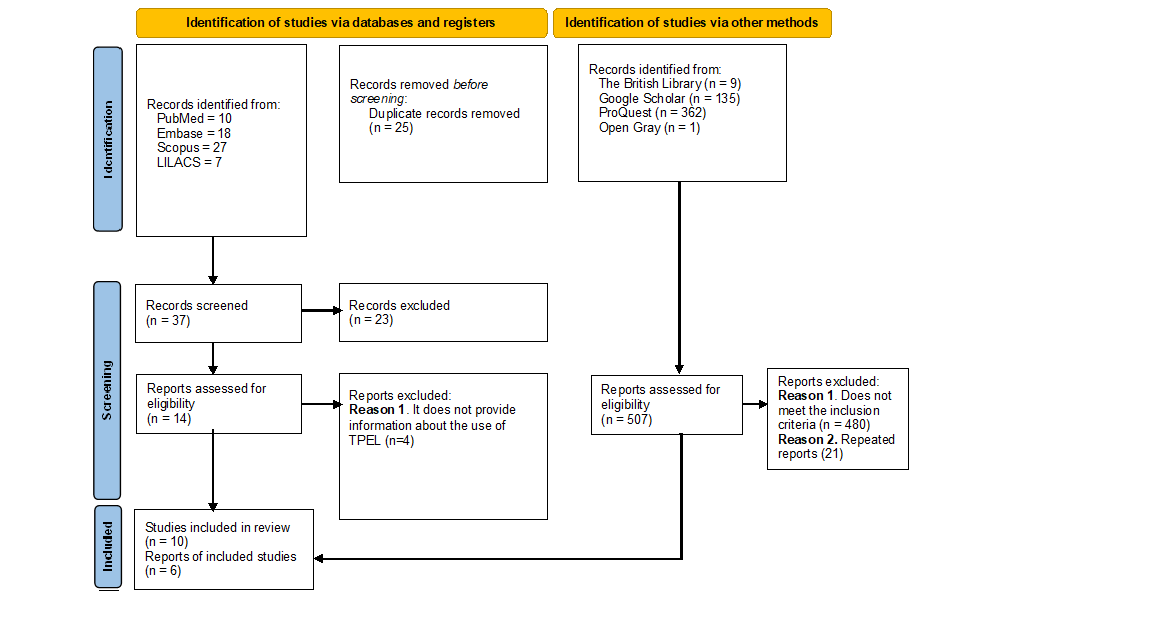

Supplement: Supplementary file 2 — Supporting Information S2 [file HPM-41-182-s001.tiff]
