# Supplementary material for: Theory of Peaceful End of Life: Analysis and Evaluation Using the Whall Framework
Source: Int J Health Plann Manage. 2025 Nov 30;41(1):182–92. doi: 10.1002/hpm.70041 (PMC12794129; doi:10.1002/hpm.70041)
Supplement: Supplementary file 3 — Supporting Information S3 [file HPM-41-182-s003.docx]

*Appendix 3. Extraction of the main characteristics of the studies included in the scoping review*

| Title | Authors | Year | Country | Journal | Study design | Aim | Use of theory | Key findings |
| --- | --- | --- | --- | --- | --- | --- | --- | --- |
| Attitude toward care of the dying and practice of peaceful  end-of-life care in community hospitals in China | Li X; Kongsuwan W; Yodchai K, et al. | 2021 | China | Internacional Nursing Review | Cross-sectional | To assess the attitude of two nurses concerning the care of dying patients and their practice of peaceful end-of-life care | Development of the instrument, Nurses' Practice of Peaceful End of Life Care Instrument (NP-PECI) according to the TPEL to assess the care at the peaceful end of life of two nurses | - The TPEL provides a theoretical reference and guidelines for the clinical practice of sickness for the quality control of non-peaceful care, thus improving the entire palliative care system.  - This study provides a new, reliable tool based on TPEL to assess nurses at their peaceful end-of-life care practice level. |
| Conforto no final de vida na terapia intensiva: percepção da equipe multiprofissional | Pires, Isabella Batista, et al. | 2020 | Brazil | Acta Paulista Enfermagem | Qualitative | To analyze the perception of the multi-professional team about comfort at the end of life in intensive care | Data analysis based on TPEL | The comfort was the concept of the TPEL that stood out in the perception of the multi-professional team, being promoted by all the categories in its care practice for patients ending up in the ICU, motivated by the identification of these basic needs, which are inserted in physical, psychological comfort, or promoted by own modification of environmental conditions. In the light of TPEL, it is noted that comfort was associated with the other concepts for promoting a peaceful end of life. |
| Cuidado espiritual prestado pela equipe de enfermagem à pessoa em  paliação na terapia intensiva | Batista VM, et al. | 2022 | Brazil | Revista Gaúcha de Enfermagem | Qualitative | To learn how the spiritual care provided by the nursing team occurs to people in palliation in the Intensive Care Unit | Data analysis based on TPEL | In light of TPEL, it is noted that comfort was associated as a possibility through spirituality and religiosity for promoting the peaceful end of life and awakening to the possibility of obtaining sick care based on theories. |
| La enfermera en los cuidados paliativos en unidades de cuidados intensivos una Teoría del Final de Vida Pacífico | Pereira, G.S | 2022 | Brazil | Revista Cubana de Enfermería | Qualitative | To understand the performance of palliative care nurses using the Theory of peaceful end of life (TPEL) | Data analysis based on TPEL | - The elements of the TPEL identified in the development of care by two nurses suggest their use in the planning and execution of the assistance services.  - The performance of two nurses assisting the patient in PC evidenced all the aspects of TPEL, emphasizing the relief of pain and better comfort. |
| Muerte y morir en el hospital: una mirada social, espiritual y ética  de los estudiantes | Sandoval, Sylvia Álvarez, et al. | 2020 | Chili | Escola Anna Nery | Qualitative | To identify the social, spiritual, and ethical implications of the death and dying process for students of  get sick | Data analysis based on TPEL | The evidence shows that this end-of-life quest can be approached differently in the classroom and practice. Likewise, innovative teaching was suggested in the theoretical classroom using TPEL, which is of simple and clear ideas, allowing easy understanding for someone and delivering tools for better care at the end of life. Furthermore, this theory guides nurses' skills to perform with two patients and their families during end-of-life care. |
| Palliative care and communication: a reflection in the light of the Peaceful End of Life Theory | De Andrade, C.G, et al. | 2022 | Brazil | Cogitare Enfermagem | Qualitative | To analyze the contribution of nursing care, with an emphasis on communication, for the patient in palliative care in the terminal phase and their relatives | Use of the TPEL to analyze the sick team's communication process with patients' relatives in palliative care in the final phase of life.  Data analysis based on TPEL | At the end of life, the patient needs to be cared for at the last moments, with dignity and a good quality of life. Therefore, this study contributed to the construction of knowledge about communication as a strategy in response to this group and the importance of the presence and dialogue of important people for the patient and family in PC. |
| Peaceful End of Life in an Unviable Newborn: A Case Report | Saldaña Agudelo G, at al. | 2020 | Colombia | Indian Journal of Palliative Care | Case report | To reflect on or care for the ultra-premature newborn | Application of nursing care to the newborn and his family according to TPEL | The TPEL is useful for generating a comprehensive neonatal or perinatal PC plan and favors evidence-based practice. |
| Promoting Peaceful Death for Thai Buddhists | Kongsuwan, W.; Touhy, T., et al. | 2009 | Thailand | Holistic Nursing Practice | Development of a model | To discover a conceptual model of a peaceful death from the perspective of two Buddhists in Thai culture | Use of theory for the development of a theory of a specific situation | This article reveals that the subconcepts of the concept of being at peace, derived from TPEL, can be modified within a Buddhist perspective. Nurses are encouraged to consider the concept of being at peace from this perspective when caring for patients who end up living in a Buddhist tradition. |
| Providing End-of-Life Care to COVID-19 Patients: The Lived  Experiences of ICU Nurses in the Philippines | Jimenez OB; Trajera SM; Ching GS | 2022 | Philippines | International Journal of Environmental Research and Public Health | Experience report | To examine the experience lived by nurses in the COVID-19 patient | The TPEL provides the basis to explain the several untold experiences encountered by ICU nurses during the COVID-19 pandemic | Using the theory of bureaucratic care and the TPEL as a framework, it is evident from the lived experience of ICU COVID-19 nurses that they clearly understand their role in facing risky encounters in the course of their duties. |
| The Effects of a Peaceful End- of-Life-Care Program on Peaceful Death as Perceived by  End-Stage Cancer Patients Receiving  Chemotherapy | Onanong W; Matchim Y, et al. | 2020 | Thailand | Science & Technology Asia | Quasi-experimental | To examine the effects of a non-life-giving peaceful care program perceived by terminal-stage cancer patients receiving chemotherapy | Development of a 4-week peaceful end-of-life care program based on TPEL | The results show that the 4-week peaceful end-of-life care program helped patients with terminal cancer receiving chemotherapy achieve a sense of peaceful death in all physical, psychological, social, and spiritual aspects. It also enhanced their perception of place and environmental characteristics before dying. |
| Contribuições da teoria final de vida pacífico para assistência de enfermagem ao paciente em cuidados paliativos | Ana Aline Lacet Zaccara, et al. | 2020 | Brazil | Revista Cuidado é Fundamental | Qualitative | To investigate the contributions of the TPEL for the assistance to patients in Palliative Care | Guide for the construction of interview questions and analysis of two data in the light of TPEL | The main contributions of the TPEL consist in providing appropriate support to guide the strategies used by nurses, especially not referring to the promotion of peace through attention to the spiritual dimension and respect for the dignity of the patient in the final phase of life-related to the care of the latter wishes of the patient and the solution of badly resolved situations. |
| Analysis of Nursing Process on Nasopharyngeal Cancer Patient with Peaceful End of Life Theory Approach: A Case Report | Endah Panca Lydia Fatma | 2022 | Indonesia | Journal of Nursing Science Update | Case report | To apply the TPEL to a patient with nasopharyngeal cancer | The patients were analyzed​​ using the Theory of a peaceful end of life. | The application of the peaceful end of life theory is appropriate for cancer patients in palliative conditions to improve their quality of life, free from pain, increasing feelings of comfort, affection and respect, peace, and sensation of closeness with people who are significant to life give them. The conclusion is that the theoretical approach focuses on the physical problems experienced and the psychological, social, and spiritual needs. |
| Cuidado à pessoa idosa institucionalizada na perspectiva de um fim de vida pacífico | Manuela Bastos Alves, et al. | 2023 | Brazil | Ciência, Cuidado e Saúde | Qualitative | To understand how care is provided at the end of life to the elderly at the *Instituição de Longa Permanência para Idosos* using the TPEL | Data analysis based on TPEL | The control of pain and physical comfort are more present in end-of-life care in ILPI, highlighting the need for greater investment in the permanent education of the team of caregivers to provide care for the elderly and their families, especially not regarding autonomy and in preparation for mourning. |
| Cuidados paliativos da enfermagem no cenário pandêmico conforme a teoria de final de vida pacífico | Jonas Melo de Matos Júnior, et al. | 2023 | Brazil | Revista Cuidado é Fundamental | Qualitative | To discover the care perspective of the nursing team in an ICU for patients diagnosed with COVID-19 and for therapeutic possibilities in light of the TPEL | Data analysis based on TPEL | The principles of humanization were consistent with the theory. However, the lack of standardization in pain assessment was a problem. The absence of significant others at the end of life made it impossible for patients with COVID-19 to experience a peaceful end. |
| Family perceptions of quality of end of life in LGBTQ+ individuals: a comparative study | S. Alexander Kemery, et al. | 2021 | USES | Palliative Care and Social Practice | Cross-sectional, comparative | To provide information to address the research gap, examining the end-of-life experiences from the perspective of two family members and comparing the experiences of LGBTQ and non-LGBTQ people in PC | The five concepts of the TPEL are linked to the items of the Questionnaire Self-applicable after the death of relatives/friends (QODD3.2-FAM) of version 3.2a of the Quality of Dying and Death (QODD). | The comparison of experiences between the cohort of lesbian, gay, bisexual, transgender, and queer individuals (n = 56) and the non-LGBTQ cohort (n = 66) yielded mixed results, with the LGBTQ cohort experiencing a lower quality of end of life on some measures of dying and death and no statistically significant difference from the non-LGBTQ cohort on others. |
| Early Initiation of Advance Care Planning for Veterans With Chronic Illnesses | Elizabeth Sipes, et al. | 2015 | USES | Federal Practitioner | Quality improvement project | To educate the PCP on the importance of allowing veterans to express their goals of care in the form of an AD and understand that veterans prefer to discuss these goals with their PCP | The indicator of TPEL – the experience of dignity/respect with its related criteria and prescribers – provides the structure for the development and implementation of this project. | Advance care planning and ADs should be a regular part of the health care process, especially for veterans with non-cancer diagnoses such as CHF and COPD. Clear communication about the trajectory and prognosis of the disease is an important part of this discussion. Primary care providers are in the optimal environment to initiate this discussion. |

TPEL- Theory of Peaceful End of Life; ICU- Intensive care unit; PC- Palliative care; PCP- Primary care provider; ADs- Advance directives; CHF- Congestive heart failure; COPD- Chronic obstructive pulmonary disease
